# Vascular Port Complication Leading to Surgery of Pulmonary Artery Branch—A Case Report

**DOI:** 10.3390/medicina60071093

**Published:** 2024-07-04

**Authors:** Dawid Kordykiewicz, Patryk Skórka, Maja Morozik, Janusz Wójcik, Małgorzata Edyta Wojtyś

**Affiliations:** Department of Thoracic Surgery and Transplantation, Pomeranian Medical University in Szczecin, Alfreda Sokołowskiego 11, 70-891 Szczecin, Poland

**Keywords:** catheter, gastric cancer, thoracotomy, complication

## Abstract

A vessel port, implanted into the central venous system, is used for long-term intravenous drug administration in oncology patients. Although essential for frequent chemotherapy and other treatments, ports can lead to complications such as infection and thrombosis. This article discusses a rare but serious complication: the displacement of a catheter fragment. A 67-year-old gastric cancer patient, experienced malignant recurrence with jaundice and bile duct infiltration post Roux-Y subtotal gastrectomy and D2 lymphadenectomy. After nine cycles of chemotherapy, a catheter fragment from the venous port detached and lodged in a branch of the pulmonary artery in segment VIII of the right lung. Thoracotomy was performed to remove the foreign body. Our aim is to report on the surgical treatment of a displaced detached catheter and to raise awareness about the potential rare complications associated with the use of vascular ports in patients undergoing chronic oncological treatment. Additionally, we screened the PubMed database for similar surgical treatment reports and compared the collected data. Venous port malfunction or non-specific patient symptoms may indicate rare complications, such as port component detachment, necessitating a multidisciplinary approach for prompt diagnosis and management in oncological patients.

## 1. Introduction

A venous access port comprises a port chamber connected to a central catheter, which is implanted into the central venous system. These ports are utilized for long-term intravenous drug administration, particularly in oncology patients who may require frequent chemotherapy, parenteral nutrition, and blood transfusions. Advancements in chemotherapy and the expanded array of available drugs have extended treatment durations and increased the frequency of doses administered. Consequently, there is a growing need for multiple ports to ensure reliable peripheral venous access. However, port installation carries a risk of complications, the most common being infection, catheter-related thrombosis, port occlusion, and intravascular dislocation [1,2,3,4]. In this article, we focus on a specific complication: the displacement of detached catheter fragments into the vascular system. Although this occurs in only 0.2–0.9% of cases, it is a potentially serious and life-threatening complication [5,6]. This study presents the case of a gastric cancer patient with a vascular port installed for chemotherapy, in whom a fragment of the catheter detached and became lodged in the pulmonary artery. We aim to detail the surgical treatment and highlight the importance of cooperation between oncologists, radiologists, and surgeons. Additionally, raising awareness among healthcare professionals about potential rare complications in chronically treated oncology patients underscores the importance of individualized therapy and may increase the effectiveness of the treatment process.

## 2. Case Presentation

**Figure 1:**
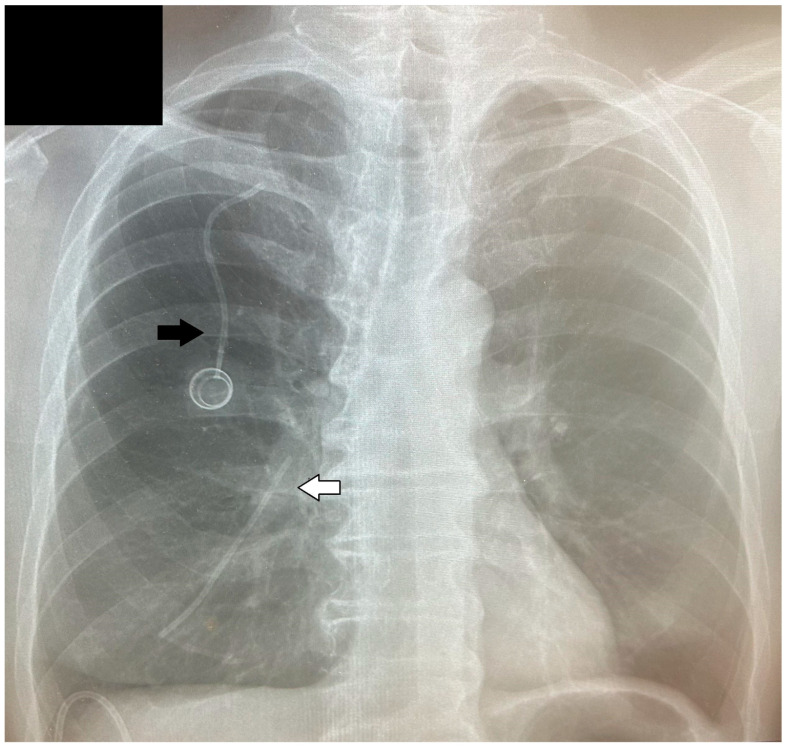
A fragment of the catheter in a pulmonary vessel (white arrow) separated from the vascular port (black arrow).

**Figure 2:**
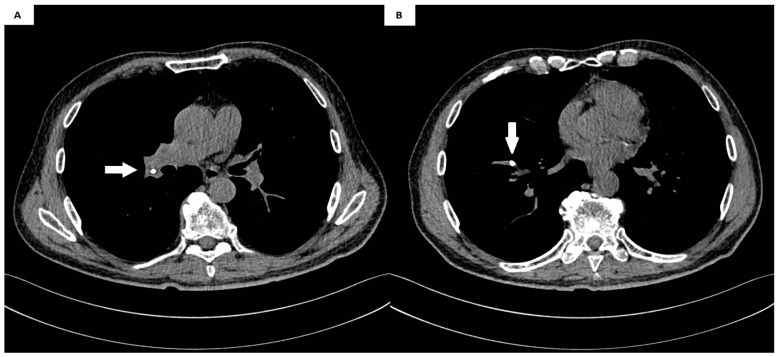
(**A**,**B**) The catheter fragment (white arrow) in the segmental artery of the right lung.

A 67-year-old male patient with stomach cancer underwent a Roux-Y subtotal gastrectomy with D2 lymphadenectomy and neoadjuvant chemotherapy. Nearly one year postoperatively, clinical manifestations indicated a malignant recurrence. The patient presented with jaundice and was admitted to the gastroenterology department, where investigations confirmed mechanical jaundice secondary to bile duct infiltration. The recurrence was characterized by anastomotic infiltration, peritoneal involvement, and lymphadenopathy in the hepatic hilum. Percutaneous drainage was performed, and the patient was referred for chemotherapy. At the beginning of August, a central vascular port (B.Braun Celsite Concept ST501) with a 20 cm catheter was inserted and chemotherapy was initiated without complications. About a month later, the patient received another dose, and after a week, the patient experienced a persistent cough, weakness, nausea, and heartburn. The patient contacted his oncology treatment center, which recommended a visit to the thoracic surgery department due to the significant symptoms of cough and chest discomfort. An X-ray in the emergency department revealed that a fragment of the catheter had separated from the port (Figure 1). Subsequent chest computed tomography (CT) imaging identified the catheter fragment lodged in the branch of the pulmonary artery in segment VIII of the right lung (Figure 2). Additionally, ultrasonography demonstrated bile accumulation in the gallbladder and intrahepatic bile duct dilation up to 6 mm. The patient was initially admitted to the thoracic department due to the presence of a foreign body in the right pulmonary artery.

**Figure 3:**
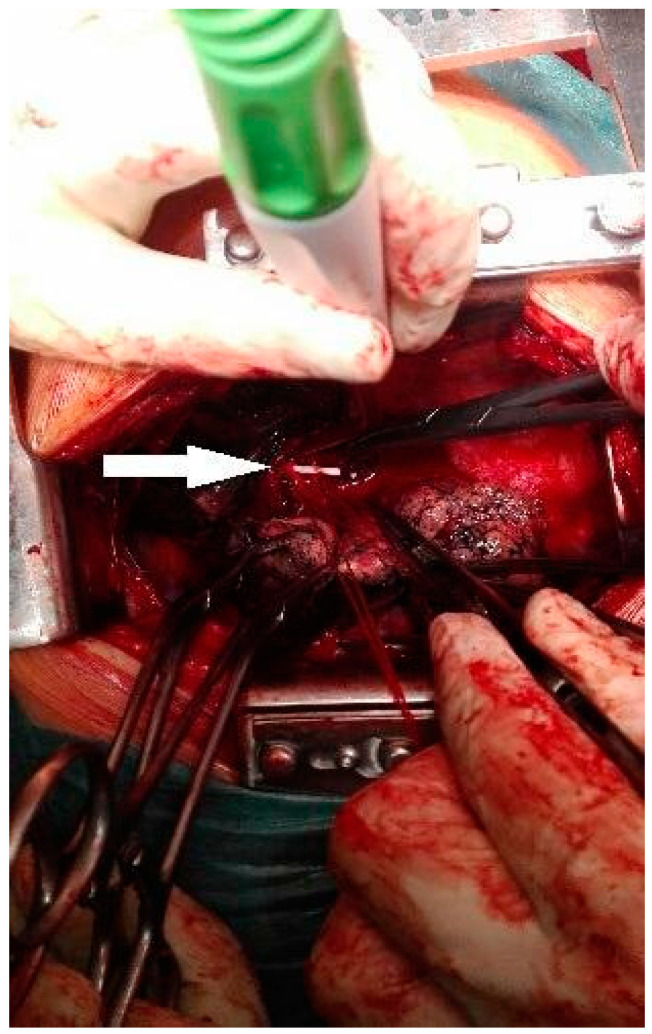
Catheter visible in the lumen of the arterial vessel (white arrow).

**Figure 4:**
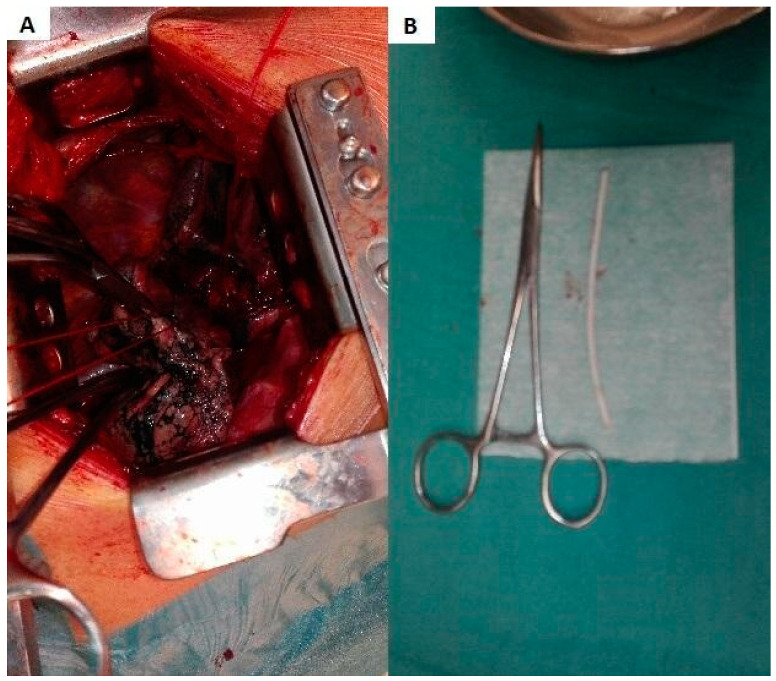
(**A**) Clamped pulmonary artery. Preparation for catheter removal. (**B**) A fragment of the catheter, approximately 8 cm in length, is shown alongside hemostat locking forceps for comparison.

A muscle-sparing thoracotomy was performed at the fifth intercostal space. The pleural cavities were clear, and the interlobar fissure was incomplete. Posterior fissure separation reconstruction was accomplished using a stapler, and the pulmonary artery wall was prepared for incision. Following the administration of 5000 units of heparin, the main trunk of the right pulmonary artery branch was clamped with a Satinsky clamp. An incision was made longitudinally in the arterial wall at the base of the interlobar fissure, and the catheter fragment was located and extracted (Figure 3 and Figure 4). The arterial wall was then closed using primary closure, the clamp was removed, and pulmonary circulation was restored. The patient’s postoperative recovery was uneventful. Subsequently, the patient was transferred to the oncology department for further management of the leaking percutaneous drainage and removal of the remaining vascular port. The patient died two months later due to the advancement of cancer.

## 3. Discussion

Venous access devices comprise a central venous catheter typically constructed from silicone or polyurethane, along with a subcutaneously implanted titanium or plastic injection port. These devices offer a simple and enduring method for accessing the vascular system to administer intravenous drugs and parenteral nutrition. A venous port catheter is subcutaneously implanted through various central veins, with the subclavian vein being typically accessible in most cases. Additionally, the catheter tip should be positioned at the junction of the superior vena cava and the right atrium under radiological guidance. Other puncture sites include the internal or external jugular veins and the common femoral vein.

Vascular catheter-related complications are classified into early complications up to 30 days after implantation and late complications occurring beyond 30 days. Initial adverse events include catheter misplacement and associated arrhythmias, perforations, and bleeding leading to hemothorax, cardiac tamponade, and pneumothorax, among others. Late adverse events include infection, pulmonary congestion, venous congestion, and catheter rupture or detachment complicated by catheter embolization [1]. The overall complication rate has been reported to range from 7.2% to 33%, with infections of the port system being the most prevalent complication [1,7]. Instances of mechanical damage to the catheter, including fractures, classified as late complications, are exceedingly rare—approximately 1.0–4.3% [8,9,10,11]. The most common causes of intravascular catheter embolization were pinch-off syndrome, failure related to time of use and proximal or distal rupture, respectively [12]. In addition, the authors highlighted that embolized fragments were most often located in the pulmonary artery. Pulmonary artery embolism caused by a catheter fragment is a very rare complication, most often asymptomatic with associated vascular port dysfunction [8]. The possible implication of pulmonary artery obstruction is local hyperperfusion with alveolar hemorrhage, and subsequent necrosis complicated by pulmonary infarction [13]. In our material, the severed catheter fragment was also located in the pulmonary artery, but the patient exhibited non-specific respiratory symptoms: a persistent cough and discomfort in the chest. In the case of an obstructed port-a-cath, immediate performance of a chest X-ray is recommended for the initial assessment of catheter displacement. Additionally, Doppler ultrasound evaluation of the major venous vessels should be conducted to rule out thrombosis [14]. Subsequently, a chest CT scan confirmed the displacement of the catheter fragment. The choice of method to remove an asymptomatic foreign body located in the cardiovascular system is dependent on the location, symptoms and risk factors [15]. Moreover, modern emphasis has been directed mainly to endovascular treatment methods for catheter embolization [16,17]. K. Wu et al. highlighted that endovascular removal is safer and simpler due to the minimal invasiveness of the procedure, especially in oncological patients [18]. Gooseneck loop snares are widely favored for endovascular retrieval of port fragments, particularly when a free end is accessible. In other cases, a pigtail catheter may be utilized to transport the dislodged fragment [19,20]. However, despite the possibilities offered by the percutaneous approach, it may still fail. In the presented case, due to the symptoms and time constraints, thoracotomy was chosen as the alternative as it was not technically possible to perform the procedure endovascularly at that time. The patient was admitted to the emergency department with respiratory symptoms, including a cough and chest discomfort, necessitating the urgent performance of a procedure before continuing treatment for the underlying condition (gastric cancer) and its complications. The appropriate positioning of the vascular catheter and the center’s expertise allowed the thoracotomy to be performed successfully and without complications. However, to the best of our knowledge, only three articles in medical databases have described the removal of a venous port fragment via surgery (Table 1).

The choice of thoracotomy as a method for removing a fragment of a catheter from the pulmonary artery is extremely rare. In the available literature, each described case resulted in the patient being discharged home with a favorable outcome. Dato et al. published a series of 14 cases in which they described foreign bodies located in the heart and pulmonary arteries. The foreign body was located in the pulmonary artery in only three cases; each patient exhibited non-specific symptoms such as anxiety or chest pain, or was asymptomatic. However, a right thoracotomy was performed only at the request of a 22-year-old patient who had a fragment of a central venous catheter in the right pulmonary artery. In this article, the authors emphasized that the location in the cardiovascular system and symptoms influence the choice of foreign body removal [15]. A comprehensive approach to such rare cases should primarily involve endovascular intervention. If this intervention is unsuccessful or vascular access is unavailable, thoracotomy becomes the primary method, as reported by Abad et al. They emphasized that this treatment regimen was most effective for patients with a catheter fragment in the right pulmonary artery [21]. Due to the anatomical structure of the lungs, the most common site of embolization is the right pulmonary artery, as seen in three cases, including the one presented here. However, Oz et al. reported an unusual location of the catheter in the left pulmonary artery [22]. In this case, the infected catheter additionally caused a pseudoaneurysm in the pulmonary artery, and the patient presented with characteristic respiratory symptoms. Due to numerous complications, immediate surgical treatment was required. In the above case, as in ours, thoracotomy was the method of choice. Several centers have also discussed alternative approaches in their articles. A review by Fisher et al. demonstrated that embolization is associated with a high mortality rate in patients from whom embolized catheter fragments were not removed [23]. The incidence of death and life-threatening complications, such as pulmonary thromboembolism, perforation and cardiac arrest, was reported to be 71%. Additionally, catheter embolism has been associated with tricuspid valve endocarditis [24]. These significant mortality and complication rates can likely be attributed to the prevalent use of rigid catheter materials during that period. At present, more advanced materials are employed for ports, and fatal outcomes are now primarily documented in isolated case reports [25]. In cases where endovascular catheter removal is unsuccessful, the patient is asymptomatic, and life expectancy is limited, treatment may involve leaving the embolized fragment in the pulmonary artery while providing medical follow-up and anticoagulant therapy. As indicated by some case reports, this approach can yield favorable long-term outcomes [26,27,28]. However, there are limited data supporting the safety and long-term effects of this management strategy. The type of recommended intervention should be considered on the basis of the patient’s current condition, the location of the catheter, and an assessment of possible complications. Cooperation between the oncologist, radiologist, and surgeon is crucial for effective intervention.

## 4. Conclusions

Venous port malfunction or non-specific patient symptoms may raise suspicion of rare complications, such as port component detachment leading to pulmonary embolism. Effective management of oncological patients necessitates a multidisciplinary approach to promptly diagnose and address such complications.

## Figures and Tables

**Table 1:** Characteristics of included patients. NR: not reported.

First Author	Sex	Age	Primary Diagnosis	Symptoms	Localization	Length of Catheter Fragment [cm]	Method
Dato [15]	M	22	NR	Anxiety	Right pulmonary artery	4	Thoracotomy
Abad [21]	M	48	Colorectal carcinoma	Asymptomatic	Right pulmonary artery	5.5	Thoracotomy
Oz [22]	M	57	Gastric malignancy	Dyspnea, chest pain, fever	Left pulmonary artery	NR	Thoracotomy
Presented case	M	67	Gastric cancer	Cough, weakness, nausea, and heartburn	Right pulmonary artery	8	Thoracotomy

## Data Availability

The data presented in this study are available in this article.
